# Cost-consciousness among Chinese medical staff: a cross-sectional survey

**DOI:** 10.1186/s12913-022-08142-8

**Published:** 2022-06-06

**Authors:** Fei Liang, Shu Hu, Youqi Guo

**Affiliations:** 1 Department of Histology and Embryology, College of Basic medicine, China Medical University, Shenyang, People’s Republic of China; 2 College of Marxism, China Medical University, Shenyang, People’s Republic of China

**Keywords:** Cost consciousness, Chinese medical staff, Uncertainty in practice

## Abstract

**Background:**

Rapidly increasing health care costs are a widespread problem in the world. The cost-consciousness among Chinese medical staff is an important topic that needs further investigation. Our study aimed to focus on the cost-consciousness of Chinese medical staff and explore the factors related to their cost-consciousness. Differences regarding cost-consciousness between doctors and nurses were also reported.

**Methods:**

Eight hospitals in Liaoning Province, China, were surveyed using a self-reporting questionnaire. A total of 1043 respondents, including 635 doctors and 408 nurses, participated in the study. A revised Chinese Cost-consciousness Scale was used to estimate cost-consciousness.

**Results:**

The mean score of the Cost-consciousness Scale was 27.60 and 28.18 among doctors and nurses, respectively, and there were no significant differences in any personal characteristics. Most Chinese medical staff were aware of the treatment costs and considered cost control as their responsibility. Chinese doctors disliked adhering to guidelines more and preferred to remain independent in making or denying a treatment decision; thus, they like autonomously balancing the treatment and cost. Chinese nurses have similar attitudes, but nurses tended to deny costly services and interventions and were more sensitive to the health care costs by rationing decisions and uncertainty in their medical practice.

**Conclusion:**

We reveal the attitudes regarding cost-consciousness among Chinese medical staff. Chinese medical staff was aware of their responsibility in health cost control. Chinese doctors and nurses had different tendencies with regard to health care cost containment. Our study highlights the importance of education and professional training on cost-consciousness.

## Background

China’s health care system has been engaged in a significant reform since 2009. Since then, the health insurance coverage rate increased to over 90%, which makes this country with a population of 1.4 billion a universal health insurance coverage country [1, 2]. Although from 2000 to 2016, total health expenditure increased nearly 10 times and governments at all levels have begun investing, Chinese health care system still received less satisfaction from all stakeholders and need further reform [3]. Meanwhile, it also raised concerns about China’s health care system’s economic sustainability. Rapidly increasing health care costs are a universal problem in the world at this time [4]. It was estimated that health system “waste” is approximately 30% of the expenditure of health care costs in the United States [4], which is economically unsustainable. The Chinese government’s health expenditure increased 3.5-fold between 2008 and 2018. Meanwhile, China has become an aging society [5]. With rapid population aging, China has to face higher demands for medical services and increased health expenditure in the future [6]. In the health care system, medical staff plays a key role in health care spending and quality. It was reported that the tendency towards excessive medical spending is due to insufficient cost-consciousness among medical staff in many countries [7–9]. The attitudes towards cost-consciousness among medical staff are important for health care cost control [10, 11].

Cost-consciousness has been defined as “the amount of attention paid to cost and an individual’s perceived responsibility to keep cost under control “ [12]. Generally, doctors play a crucial role in medical resource distribution and cost containment. Doctors are involved in approximately 80% of the healthcare expenditure decisions [13]. Most doctors also regard cost-containment as their responsibility [11, 12]. These decisions regarding medical tests and treatments can be influenced by personal characteristics [14], education [15], work context [12], and tolerance for uncertainty [11, 16]. In addition, nurses’ cost-consciousness should also not be overlooked. As the staff working in the same place as doctors, nurses’ opinions about cost-consciousness might influence the doctors’ perceived responsibility regarding health expenditure control. The economic awareness of nurses is also one of the major cost drivers of clinical care [8, 17]. Nursing care, such as added consumables, is also important for daily routine costs [12]. Except for health cost expenditure, greater cost-consciousness was associated with fewer low-value clinical services [18]. The cost-consciousness among medical staff is a noteworthy matter for public health policymakers and hospital managers alike.

However, to our knowledge, there are no studies focusing on the cost-consciousness of Chinese medical staff. Our study, therefore, aimed to focus on this important but under-explored topic and explores the factors related to cost-consciousness. We also compared the differences in cost-consciousness between Chinese doctors and nurses.

## Methods

### Study design and participants

The study of cost-consciousness is part of a pen-and-paper questionnaire survey that was conducted from September 2017 to January 2018 in Liaoning Province in northeastern China. Geographically, Liaoning Province can be divided into three regions: the western, central, and southern regions. According to the geographical distribution, we selected three representative cities in each region and two to three general tertiary hospitals from each city. Thus, doctors and nurses from a total of eight hospitals were invited to participate in the survey. We received assistance from the administrators and medical staff of all hospitals during the questionnaire survey. Eventually, we obtained 1070 completed questionnaires. The factors and scales included demographic information (gender, age, marital status, and education), work conditions (position, professional title, monthly income, and working hours), cost-consciousness, and uncertainty in practice.

### Measurements

#### Cost-consciousness scale

Cost-consciousness was measured using a revised Chinese version of the Cost-consciousness Scale (CCS). The revised CCS was based on Tilburt et al.’s study [10]. In this study, Tilburt et al. surveyed US doctors’ professional role in cost-containment and derived an 11-item cost-consciousness scale from an original 13 items. These 13 items included a two-item Stewardship Scale [10], the three-item Agreement with Rationing Scale [19], the six-item Cost-Consciousness Scale [20], and the remaining two items were developed by Tilburt et al. [10]. We selected 11 items from the original 13 items to create the revised CCS. The revised CCS has two different items compared to Tilburt et al.’s 11-item scale to better fit the circumstances in China. We replaced “doctors” in the items with “doctors/nurses” in order to address both doctors and nurses. A four-point Likert scale was used to score the items of the instrument (from 1 for “strongly disagree” to 4 for “strongly agree”). In the revised Chinese version 11-item CCS, two items come from a Stewardship Scale (Q1, Q2) [10], and three items come from the three-item Agreement with Rationing Scale (Q3, Q4, Q5) [19]. Another five items belong to the six-item Cost-consciousness Scale (Q6–10) [20], of which two items (Q6, Q7) explore opinions regarding health care costs and the other three items (Q8, Q9, Q10) are about attitudes regarding the costs of tests and procedures [11]. Q11 was developed by Tilburt et al. to measure the consideration of cost when making treatment decisions [10]. Higher scores on the scale reflect a greater degree of cost-consciousness. The detailed CCS is shown in Table 3. The Cronbach’s alpha of the scale in our study is 0.77.

#### Uncertainty in practice

Uncertainty is common in the practice of medicine [21]. In previous studies, the attitude toward uncertainty in practice was regarded as a possible barrier to influence resource utilization [10, 20]. We used two questions measured on a four-point Likert scale ranging from “strongly disagree” to “strongly agree” to evaluate uncertainty in practice [10].

#### The translation stage

The translation was performed as follows. Two researchers translated all items from English to Chinese independently, and an agreement was reached through discussions on the translation. Then, the Chinese version was reverse-translated into English. The differences in the translations were discussed and resolved with all researchers [22].

### Statistical analysis

All data from the questionnaires were input in EpiData 3.0 software. The statistical analysis was performed by SPSS version 25.0 (IBM, Armonk, NY, USA). All statistical tests were two-tailed, and *p* < 0.05 was considered significant.

Student’s *t* test, a one-way analysis of variance, was performed to examine the difference in the CCS according to personal and work characteristics among doctors and nurses. The scores of the CCS were treated as continuous variables.

Student’s *t* test was performed to examine the difference in the CCS between doctors and nurses. The scores of the CCS and uncertainty were treated as continuous variables.

We performed a hierarchical linear regression analysis for the CCS. Variables, including demographics (age, gender, education, and marital status) and work situations (professional title, monthly income, working hours), as well as uncertainty in practice, were entered into the model.

The study was checked by a STROBE checklist of items for a cross-sectional study.

## Results

We distributed approximately 1200 questionnaires to medical staff. After excluding surveys with missing information (gender, age, marital status, education, or work conditions), the final total of 1043 respondents included 635 doctors and 408 nurses who became the study subjects. The response rate of this study was 86.9%.The demographic and work condition characteristics of the respondents in the survey are shown in Table 1. In this survey, 46.0% of the doctors were male, but 97.8% of the nurses were female. Most respondents were less than 40 years old (67.4% of doctors and 74.3% of nurses), and most of the respondents were married. The doctors had a higher education level: 95.7% of doctors had a bachelor’s or a higher degree, but more than half of the nurses had a bachelor’s or a lower degree. The percent of different professional titles (junior/middle/senior) among doctors were 38.9, 33.2, and 27.9%, respectively, and only 14 nurses (3.4%) had a senior title. Most doctors and nurses reported that their monthly incomes were between 3000 and 8000 CNY (about 450 to 1200 USD). The doctors had longer working times, and nearly 60% reported that they worked more than 50 hours per week, compared with 83.3% of nurses who worked less than 50 hours per week.

**Table 1:** Personal characteristics of the Survey

	Doctors ***N*** = 635 (%)	Nurses ***N*** = 408 (%)
**Gender**		
**Male**	292 (46.0)	9 (2.2)
**Female**	343 (54.0)	399 (97.8)
**Age(y)**		
**< 30**	117 (18.4)	135 (33.1)
**30–39**	311 (49.0)	168 (41.2)
**40–49**	138 (21.7)	65 (15.9)
**≥ 50**	69 (10.9)	40 (9.8)
**Marital status**		
**Single**	158 (24.9)	126 (30.9)
**Married**	467 (73.5)	276 (67.6)
**Divorced or widowed**	10 (1.6)	6 (1.5)
**Education**		
**College degree or lower**	27 (4.3)	221 (54.2)
**Bachelor degree**	338 (53.2)	186 (45.6)
**Master‘s degree or higher**	270 (42.5)	1 (0.2)
**Professional Title**		
**Junior**	247 (38.9)	248 (60.8)
**Middle**	211 (33.2)	146 (35.8)
**Senior**	177 (27.9)	14 (3.4)
**Income per month (CNY)**		
**< 3000**	91 (14.3)	132 (32.4)
**3000-4999**	270 (42.5)	175 (42.9)
**5000-7999**	228 (35.9)	96 (23.5)
**> 8000**	46 (7.2)	5 (1.2)
**Working Hours Per Week (h)**		
**< 40**	54 (8.5)	71 (17.4)
**40–49**	210 (33.1)	269 (65.9)
**50–60**	190 (29.9)	46 (11.3)
**> 60**	181 (28.5)	22 (5.4)

The CCS score comparisons according to personal characteristics are shown in Table 2. It was unexpected that there were no significant differences in the CCS among any of the factors. It seems that neither education nor professional experience has a strong impact on cost-consciousness as the attitudes towards cost-consciousness among Chinese medical staff are stable.

**Table 2:** The scores of Cost-consciousness scale according to personal and work characteristics

	Doctors (*N* = 635)			Nurses (*N* = 408)		
Factors	Mean	SD	P	Mean	SD	P
**Gender**			**0.759**			**0.710**
Male	27.54	5.19		28.89	3.26	
Female	27.65	4.29		28.19	5.72	
**Age(y)**			**0.697**			**0.062**
< 30	27.28	3.97		27.36	6.30	
30–39	27.66	4.56		28.18	5.38	
40–49	27.49	4.82		28.89	5.45	
≥ 50	28.10	6.22		29.88	6.69	
**Marital status**			**0.293**			**0.196**
Single	27.63	4.32		27.46	6.13	
Married	27.54	4.82		28.48	5.54	
Divorced or widowed	29.90	5.80		29.83	4.58	
**Education**			**0.858**			**0.201**
College degree or lower	27.96	6.21		28.02	5.69	
Bachelor degree	27.65	4.99		28.32	5.75	
Master’s degree or higher	27.50	4.18		38.00		
**Professional Title**			**0.705**			**0.398**
Junior	27.65	4.17		27.90	5.84	
Middle	27.74	4.81		28.55	5.61	
Senior	27.36	5.31		29.43	4.73	
**Income per month (CNY)**			**0.776**			**0.085**
< 3000	27.33	4.26		28.75	6.47	
3000-4999	27.51	4.68		28.04	5.23	
5000-7999	27.71	4.60		27.43	5.49	
> 8000	28.13	6.27		33.00	2.12	
**Working Hours Per Week (h)**			**0.315**			**0.155**
< 40	27.65	6.75		28.69	6.42	
40–49	27.98	4.76		28.40	5.69	
50–60	27.66	4.06		26.54	4.68	
> 60	27.08	4.57		27.32	5.39	

The differences were examine by Student’s *t* test and ANOVA

The degrees of disagreement/agreement among respondents regarding cost-consciousness for each item are shown in Table 3. Most of the medical staff reported being “aware of the costs of the tests/treatments they recommend” (84.7% of doctors and 78% of nurses), agreed that “trying to contain costs is the responsibility of every doctor/nurse” (75.9% of doctors and 70.5% of nurses). Most of the doctors (72.4%) and nurses (66.1%) also agreed with the statement, “I should sometimes deny beneficial but costly services to certain patients because resources should go to other patients that need them more.” Most of the respondents (78.6% of doctors and 60.7% of nurses) disagreed that they “should adhere to clinical guidelines” and opposed the statement, “I should be solely devoted to my individual patients’ best interests, even if that is expensive” (64% of doctors and 57.3% of nurses, respectively). In addition, 73.5% of doctors and 70.5% of nurses disagreed with the statement that “the cost of a test or medication is only important if the patient has to pay for it out of pocket.” The differences between doctors and nurses (Table 3) were also analyzed, and we found that there are significant differences among five items (Q1, Q3, Q5, Q8 and Q9). These items are about stewardship (Q1), agreement with rationing (Q3, Q5) and attitudes regarding the costs of tests and procedures (Q8, Q9).

**Table 3:** Degree of Agreement/Disagreement among medical staff regarding Cost-consciousness scale and Uncertainty in practice

	Doctors (***N*** = 635) No.(%)					Nurses (***N*** = 408) No.(%)				
	Strongly Disagree	Moderately Disagree	Moderately Agree	Strongly Agree	Mean (±SD)	Strongly Disagree	Moderately Disagree	Moderately Agree	Strongly Agree	Mean (±SD)
**Q1. I am aware of the costs of the tests/treatments I recommend**	**17 (2.7)**	**80 (12.6)**	**314 (49.4)**	**224 (35.3)**	3.17 ± 0.75**	**26 (6.4)**	**64 (15.7)**	**208 (51.0)**	**110 (27.0)**	**2.99 ± 0.83**
**Q2. I try not to think about the cost to the health care system when making treatment decisions**	**114 (18.0)**	**300 (47.2)**	**175 (27.6)**	**46 (7.2)**	**2.24 ± 0.83**	**79 (19.4)**	**157 (38.5)**	**124 (30.4)**	**48 (11.8)**	**2.35 ± 0.92**
**Q3. I should sometimes deny beneficial but costly services to certain patients because resources should go to other patients that need them more**	**64 (10.1)**	**175 (27.6)**	**271 (42.7)**	**125 (19.7)**	2.72 ± 0.89**	**31 (7.6)**	**107 (26.2)**	**167 (40.9)**	**103 (25.2)**	**2.84 ± 0.89**
**Q4. Cost to society is important in my decisions to use or not to use an intervention**	**76 (12.0)**	**237 (37.3)**	**264 (41.6)**	**58 (9.1)**	**2.48 ± 0.82**	**49 (12.0)**	**146 (35.8)**	**164 (40.2)**	**49 (12.0)**	**2.52 ± 0.86**
**Q5. Doctors/nurses should adhere to clinical guidelines that discourage the use of interventions that have a small proven advantage over standard interventions but cost much more**	**103 (16.2)**	**333 (52.4)**	**178 (28.0)**	**21 (3.3)**	2.18 ± 0.74**	**52 (12.7)**	**196 (48.0)**	**121 (29.7)**	**39 (9.6)**	**2.36 ± 0.82**
**Q6. The cost of a test or medication is only important if the patient has to pay for it out of pocket**	**176 (27.7)**	**291 (45.8)**	**138 (21.7)**	**30 (4.7)**	**2.03 ± 0.83**	**116 (28.4)**	**171 (41.9)**	**90 (22.5)**	**29 (7.1)**	**2.08 ± 0.89**
**Q7. Doctors/nurses are too busy to worry about costs of tests and procedures**	**153 (24.1)**	**275 (43.3)**	**168 (26.5)**	**39 (6.1)**	**2.15 ± 0.85**	**75 (18.4)**	**194 (47.5)**	**100 (24.5)**	**39 (9.6)**	**2.25 ± 0.87**
**Q8.Trying to contain costs is the responsibility of every doctor/nurse**	**70 (11.0)**	**147 (23.1)**	**297 (46.8)**	**121 (19.1)**	2.74 ± 0.89**	**30 (7.4)**	**88 (21.6)**	**190 (46.0)**	**100 (24.5)**	**2.88 ± 0.86**
**Q9. There is currently too much emphasis on costs of tests and procedures**	**46 (7.2)**	**173 (27.2)**	**291 (45.8)**	**125 (19.7)**	2.78 ± 0.84*	**38 (9.3)**	**127 (31.1)**	**175 (42.9)**	**68 (16.7)**	**2.67 ± 0.86**
**Q10. Doctors/nurses need to take a more prominent role in limiting use of unnecessary tests**	**55 (8.7)**	**154 (24.3)**	**274 (43.1)**	**152 (23.9)**	**2.82 ± 0.89**	**30 (7.4)**	**93 (22.8)**	**184 (45.1)**	**101 (24.8)**	**2.87 ± 0.87**
**Q11. I should be solely devoted to my individual patients’ best interests, even if that is expensive**	**104 (16.4)**	**302 (47.6)**	**176 (27.7)**	**53 (8.3)**	**2.28 ± 0.84**	**58 (14.2)**	**184 (45.1)**	**121 (29.7)**	**45 (11.0)**	**2.38 ± 0.86**
**Uncertainty in practice**										
**1 I find the uncertainty involved in patient care disconcerting**	**35 (5.5)**	**141 (22.2)**	**351 (55.3)**	**108 (17.0)**	2.16 ± 0.77*	**27 (6.6)**	**124 (30.4)**	**188 (46.1)**	**69 (16.9)**	**2.27 ± 0.82**
**2 I generally order more tests when I don’t know the patient well**	**67 (10.6)**	**282 (44.4)**	**225 (35.4)**	**61 (9.6)**	**2.56 ± 0.81**	**59 (14.5)**	**170 (41.7)**	**136 (33.3)**	**43 (10.5)**	**2.60 ± 0.86**

Strongly Disagree = 1; Moderately Disagree = 2; Moderately Agree = 3; Strongly Agree = 4

The differences were examine by Student’s *t* test

**p < 0.01

*p < 0.05

As shown in Table 4, the mean scores of the CCS among doctors and nurses are 27.60 and 28.18, respectively, which are close to the midpoint of the CCS (27.5). The mean score of uncertainty in practice is 4.72. There are no significant differences of the CCS and uncertainty between doctors and nurses.

**Table 4:** The mean scores of Cost-consciousness scale and Uncertainty in practice

		Doctor (*N* = 635)		Nurse (*N* = 408)		
Scales	Range	Mean	SD	Mean	SD	P
**Cost-consciousness**	11–44	27.60	4.72	28.18	5.72	0.085
**Uncertainty in practice**	2–8	4.72	1.29	4.87	1.38	0.082

The differences were examine by Student’s *t* test

To analyze the effects of different variables on the CCS, we performed a linear regression analysis for the CSS. The results are shown in Table 5. Demographics and work condition variables explained 1.2 and 4.1% of the variance in the CCS and uncertainty in practice was responsible for 14.8 and 30.7% variance in doctors and nurses, respectively. The linear regression analysis showed that uncertainty has a stronger impact on the CCS among nurses.

**Table 5:** The linear regression analysis results for Cost-consciousness

	Doctors (*N* = 635)		Nurse (*N* = 408)	
Variables	Step 1(β)	Step 2(β)	Step 1(β)	Step 2(β)
**Gender**	0.007	0.004	−0.013	−0.021
**Age**	0.091	0.064	0.177*	0.147*
**Marital status**	0.015	0.077	0.003	−0.013
**Education**	−0.009	0.012	0.077	0.051
**Professional Title**	−0.122*	−0.128*	− 0.001	0.015
**Income**	0.053	0.082	0.129*	−0.029
**Working Hours**	−0.053	−0.056	0.080	−0.048
**Uncertainty in practice**		0.390**		0.564**
**F**	1.078	14.845**	2.466*	26.720**
**R**^**2**^	0.012	0.159	0.041	0.349
**ΔR**^**2**^		0.148		0.307

*p < 0.05

**p < 0.01

**ΔR**^**2**^: **R**^**2**^ increase

In step 1, Gender, Age, Marital status, Professional Title, Income and Working Hours were added

In step 2, Uncertainty in practice was added

## Discussion

Health care expenditures in China will continue to increase due to universal health insurance and rapid population aging in the future [1, 5]. As the healthcare professionals play an important role in health care expenditures [23], the cost-consciousness of medical staff is likely to significantly affect the cost being economically sustainable. To our knowledge, our study is the first one on the status of cost-consciousness among Chinese medical staff. Our results reveal the attitudes of Chinese medical staff regarding their role in containing health care costs. Studies of cost-consciousness among doctors and nurses are rare. Bovier et al. surveyed cost-consciousness using a six-item scale with 1184 Swiss doctors; however, their survey was conducted more than 20 years ago and did not include nurses [11]. Another German study used a similar six-item scale with 496 physicians and 1406 nurses; however, all of the participants came from neonatal intensive care units [12]. Tilburt et al. conducted cross-sectional surveys using an 11-item scale with US physicians in 2012 [10] and 2017 [24], respectively, and found that physicians’ attitudes toward their role in containing costs were similar in 2012 and 2017. There are some other studies about cost-consciousness [23, 25]; however, the cost-consciousness in these studies refers to the knowledge of health care or medicine costs, not the attitude toward them.

In this study, the Cronbach’s alpha score of the revised Chinese Cost-consciousness Scale is 0.77, which is similar to Tilburt et al.’s study (0.77) [10], and higher than the six-item cost-consciousness scale used in the study with Swiss doctors (0.68) [11] and the one used with German neonatal intensive care units (0.62) [12]. Thus, the revised CCS is a reliable measurement for cost-consciousness. The median score of the CCS in our study is 28, which it is lower than that found among US physicians (median score = 31) [10, 18]. Although the items were not exactly the same, it seems that Chinese medical staff had a lower degree of cost-consciousness.

The attitudes of cost-consciousness among Chinese doctors are complex. A majority of doctors agreed that being aware of the costs of tests and treatments and considering costs control is their responsibility. This is similar to the findings of previous studies conducted in Western countries [10, 11, 23, 24]. Chinese doctors have similar attitudes of cost containment to Swiss and German doctors [11, 23], except that only a small part of German doctors agree that there is too much emphasis on the costs of tests and procedures when most Chinese doctors agreed (28.23% Vs 75.5%) [23]. As our survey is most similar to Tilburt et al.’s study [10], we compared the cost-consciousness of Chinese and US doctors and found that Chinese/US doctors had different opinions regarding some items. Most Chinese doctors (78.6%) disagreed with the notion that they “should adhere to clinical guidelines that discourage the use of interventions that have a small proven advantage over standard interventions but cost much more” (Q5), but 79% of US physicians agreed with the statement [10]. Most of the Chinese doctors (72.4%) agreed with the statement that they “should sometimes deny beneficial but costly services to certain patients because resources should go to other patients that need them more” (Q3); however, 85% of US physicians disagreed with this [10]. In addition, 85% of US physicians agreed with the notion that “the cost of a test or medication is only important if the patient has to pay for it out of pocket” (Q6), while 73.5% of Chinese doctors disagreed with it [10]. Also, 65% of Chinese doctors disagreed, and 78% of US physicians agreed with the statement, “I should be solely devoted to my individual patients’ best interests, even if that is expensive” (Q11) [10]. Further, 75.5% of Chinese doctors agreed, and 66% of US physicians disagreed with the statement that “there is currently too much emphasis on costs of tests and procedures” (Q9) [10].

Considering the differences and medical circumstance disparity between China and the US, it was not surprising that there were distinct opinions between Chinese and US doctors. Generally, like US physicians, Chinese doctors also realized their responsibility for controlling healthcare costs. However, Chinese doctors more strongly disliked adhering to guidelines (Q5), more strongly preferred to independently make or deny a decision regarding treatment (Q3 and Q6), and more strongly preferred autonomously judging the balance between treatment and costs (Q9 and Q11). It is partly reasonable that Chinese doctors distrusted their guidelines due to a lack of a demonstration of transparency and quality by most Chinese guidelines [26]. However, this means that they are more willing to depend on their personal experiences and feelings, which could be more disadvantageous for providing high-quality health care and enacting cost containment. It should also be pointed out that economic motivations are widely existing among Chinese medical staff, which encourages them to provide excuses for economically motivated corruption to supplement their low incomes [27, 28]. They may, thus, be prone to extend their discretion in practice and rationalize their benefits from corruption. Therefore, Chinese doctors preferred to make treatment decision independently and balance treatment and costs autonomously, while they dislike guidelines. Compared with Chinese doctors, US physicians suffered less stress due to costs and preferred to stand individual patient interests in treatment.

Chinese nurses have similar views regarding cost-consciousness as doctors. In our study, there is no significant difference in the CCS, on the whole, between doctors and nurses. However, they had different tendencies regarding certain items. It seems that doctors know more about treatment costs and are more like to emphasize costs, while nurses more strongly prefer to deny costly services and interventions and are more sensitive to their cost-related responsibility and rationing decisions. It is not unexpected that doctors know more about the costs of tests and treatments and dislike outside interventions interfering with their practice discretions. Chinese doctors did not experience a lot of stress related to cost-containment as a result of their professional role. Meanwhile, nurses frequently face complaints and dissatisfaction related to the health care costs for the patient but do not directly benefit from the doctor’s rationing decisions. Thus, nurses were more sensitive to the health care costs and rationing decisions. This can also explain why uncertainty in practice was responsible for a much proportion of the variance of cost-consciousness among nurses (30.7%) compared with doctors (14.8%). Unexpectedly, there were no significant differences in the CCS among any factors according to demographic and work condition characteristics. Cost-consciousness was common among Chinese medical staff. This also implies that neither professional education nor clinical practice strongly impacted their cost-consciousness. It is a widespread problem that medical residents have poor knowledge of health care costs during their training [9, 25]. To improve cost-consciousness among Chinese medical staff, stronger management interventions and compulsory professional training throughout their career are necessary.

Generally, although Chinese medical staff realize their responsibility for cost-consciousness, training for cost containment is still necessary. The neglect of cost-consciousness in training can lead to a lack of knowledge of the costs of treatments or consumable items [29]. Chinese medical staff needs more training on evidence-based decision-making, as well as greater knowledge of health economics and the cost of health care. There is a clear responsibility of doctors and nurses as part of their professional roles for cost control, which should also be included in the health care system reform. This is important for providing high-value, cost-conscious care [30].

Several limitations of this study should be mentioned. First, as a self-reported questionnaire survey, respondents may distort their views to meet assumed expectations. Second, we used a revised Cost-consciousness Scale derived from studies conducted in the US. Given the different health care systems and contextual cultural disparity, this may reduce the validity of our results. Finally, the Cost-consciousness Scale was initially designed for physicians, and the revision, including both doctors and nurses, may distort their opinions about their professional roles.

## Conclusion

We performed a survey on the cost-consciousness among Chinese medical staff. Chinese medical staff was aware of their responsibility for health cost control, and Chinese doctors and nurses had different tendencies regarding health care cost containment. Our study reveals the attitudes of cost-consciousness among Chinese medical staff and highlights the importance of education and professional training to change the behavior of healthcare professionals.

## Data Availability

The datasets used and analyzed during the current study are available from the corresponding author upon reasonable request.

## Abbreviations

CCS: Cost-consciousness scale

## References

[1] Ta Y, Zhu Y, Fu H (2019). Trends in access to health services, financial protection and satisfaction between 2010 and 2016: has China achieved the goals of its health system reform?. Soc Sci Med.

[2] Sun Y, Gregersen H, Yuan W (2017). Chinese health care system and clinical epidemiology. Clin Epidemiol.

[3] Hu K (2021). Challenges facing the Chinese health care system. Asia Pac J Public Health.

[4] Hood VL, Weinberger SE (2012). High value, cost-conscious care: an international imperative. Eur J Intern Med.

[5] Li L, Du T, Hu Y (2020). The effect of population aging on healthcare expenditure from a healthcare demand perspective among different age groups: Evidence from Beijing City in the People's Republic of China. Risk Manag Healthc Policy.

[6] Lopreite M, Zhu Z (1982). The effects of ageing population on health expenditure and economic growth in China: a Bayesian-VAR approach. Soc Sci Med.

[7] Blendon RJ, Benson JM, Botta MD, Zeldow D, Kim MK (2012). A four-country survey of public attitudes towards restricting healthcare costs by limiting the use of high-cost medical interventions. BMJ Open.

[8] Jakovljevic M, Vukovic M, Chen C-C, Antunovic M, Dragojevic-Simic V, Velickovic-Radovanovic R, Djendji MS, Jankovic N, Rankovic A, Kovacevic A (2016). Do health reforms impact cost consciousness of health care professionals? Results from a nation-wide survey in the Balkans. Balkan Med J.

[9] McGuire C, King S, Roche-Nagle G, Barry MC (2009). Doctors’ attitudes about prescribing and knowledge of the costs of common medications. Ir J Med Sci.

[10] Tilburt JC, Wynia MK, Sheeler RD, Thorsteinsdottir B, James KM, Egginton JS, Liebow M, Hurst S, Danis M, Goold SD (2013). Views of US physicians about controlling health care costs. JAMA.

[11] Bovier PA, Martin DP, Perneger TV (2005). Cost-consciousness among Swiss doctors: a cross-sectional survey. BMC Health Serv Res.

[12] Schmitz H, Martakis K, Roth B, Pfaff H, Scholten N (2019). Differences in cost consciousness between physicians and nurses in German neonatal intensive care units. Acta Paediatr.

[13] Bulger J, Nickel W, Messler J, Goldstein J, O'Callaghan J, Auron M, Gulati M (2013). Choosing wisely in adult hospital medicine: five opportunities for improved healthcare value. J Hosp Med.

[14] Halm EA, Causino N, Blumenthal D (1997). Is gatekeeping better than traditional care? A survey of physicians’ attitudes. JAMA.

[15] Allan GM, Lexchin J, Wiebe N (2007). Physician awareness of drug cost: a systematic review. PLoS Med.

[16] Allison JJ, Kiefe CI, Cook EF, Gerrity MS, Orav EJ, Centor R (1998). The association of physician attitudes about uncertainty and risk taking with resource use in a Medicare HMO. Med Decis Mak.

[17] Caroselli C (1996). Economic awareness of nurses: relationship to budgetary control. Nurs Econ.

[18] Grover M, Abraham N, Chang YH, Tilburt J (2016). Physician cost consciousness and use of low-value clinical services. J Am Board Fam Med.

[19] Hurst SA, Slowther AM, Forde R, Pegoraro R, Reiter-Theil S, Perrier A, Garrett-Mayer E, Danis M (2006). Prevalence and determinants of physician bedside rationing: data from Europe. J Gen Intern Med.

[20] Goold SD, Hofer T, Zimmerman M, Hayward RA (1994). Measuring physician attitudes toward cost, uncertainty, malpractice, and utilization review. J Gen Intern Med.

[21] Gerrity MS, DeVellis RF, Earp JA (1990). Physicians' reactions to uncertainty in patient care. A new measure and new insights. Med Care.

[22] Sousa VD, Rojjanasrirat W (2011). Translation, adaptation and validation of instruments or scales for use in cross-cultural health care research: a clear and user-friendly guideline. J Eval Clin Pract.

[23] Wei D, Osman C, Dukhovny D, Romley J, Hall M, Chin S, Ho T, Friedlich PS, Lakshmanan A (2016). Cost consciousness among physicians in the neonatal intensive care unit. J Perinatol.

[24] Warsame R, Riordan L, Jenkins S, Lackore K, Pacyna J, Antiel R, Beebe T, Liebow M, Thorsteinsdottir B, Grover M (2020). Responsibilities, strategies, and practice factors in clinical cost conversations: a US physician survey. J Gen Intern Med.

[25] Maghbouli N, Akbari Sari A, Asghari F (2020). Cost-consciousness among Iranian internal medicine residents. Med Teach.

[26] Yang K, Chen Y, Li Y, Schünemann HJ (2013). Editorial: can China master the guideline challenge?. Health Res Policy Syst.

[27] Chen XY (2007). Defensive medicine or economically motivated corruption? A confucian reflection on physician care in China today. J Med Philos.

[28] Yip WC, Hsiao W, Meng Q, Chen W, Sun X (2010). Realignment of incentives for health-care providers in China. Lancet (London, England).

[29] Geoghegan AR, Moore S, O'Donnell CP (2015). Doctors’ perceptions of the cost of consumable items used in neonatal intensive care. Acta paediatr (Oslo, Norway: 1992).

[30] Stammen LA, Stalmeijer RE, Paternotte E, Oudkerk Pool A, Driessen EW, Scheele F, Stassen LP (2015). Training physicians to provide high-value, cost-conscious care: a systematic review. JAMA.

